# Health literacy strengths and challenges among residents of a resource-poor village in rural India: Epidemiological and cluster analyses

**DOI:** 10.1371/journal.pgph.0001595

**Published:** 2023-02-17

**Authors:** Reetu Passi, Manmeet Kaur, P. V. M. Lakshmi, Christina Cheng, Melanie Hawkins, Richard H. Osborne

**Affiliations:** 1 Department of Community Medicine and School of Public Health, Post-Graduate Institute of Medical Education and Research, Chandigarh, India; 2 Centre for Global Health and Equity, School of Health Sciences, Swinburne University of Technology, Melbourne, Australia; Egas Moniz Cooperativa de Ensino Superior CRL, PORTUGAL

## Abstract

Cluster analysis can complement and extend the information learned through epidemiological analysis. The aim of this study was to determine the relative merits of these two data analysis methods for describing the multidimensional health literacy strengths and challenges in a resource poor rural community in northern India. A cross-sectional survey (N = 510) using the Health Literacy Questionnaire (HLQ) was undertaken. Descriptive epidemiology included mean scores and effect sizes among sociodemographic characteristics. Cluster analysis was based on the nine HLQ scales to determine different health literacy profiles within the population. Participants reported highest mean scores for Scale 4. Social support for health (2.88) and Scale 6. Ability to actively engage with healthcare professionals (3.66). Lower scores were reported for Scale 3. Actively managing my health (1.81) and Scale 8. Ability to find good health information (2.65). Younger people (<35 years) had much higher scores than older people (ES >1.0) for social support. Eight clusters were identified. In Cluster A, educated younger men (mean age 27 years) reported higher scores on all scales except one (Scale 1. Feeling understood and supported by a healthcare professional) and were the cluster with the highest number (43%) of new hypertension diagnoses. In contrast, Cluster H also had young participants (mean age 30 years) but with low education (72% illiterate) who scored lowest across all nine scales. While epidemiological analysis provided overall health literacy scores and associations between health literacy and other characteristics, cluster analysis provided nuanced health literacy profiles with the potential to inform development of solutions tailored to the needs of specific population subgroups.

## Introduction

Common to health literacy research is descriptive epidemiological analysis where average population scores are used to estimate associations between health literacy and a range of sociodemographic variables, such as education, age, gender, and cultural background [1]. However, average population scores do not reveal the diversity of individuals or different groups within a population [2]. When the focus of data interpretation is on average population scores, a one-size-fits-all approach will be applied to public health, education, and communication initiatives, which leads to limited or no attention given to the needs of groups of people outside the norm. One-size-fits-all initiatives tend not to cater for diversity and may increase the health equity divide [3]. Complementary to epidemiological analyses are methods that can reveal patterns in health literacy differences and the sociodemographic characteristics of groups of people within populations. Identification of these patterns enables public health and health service initiatives to be developed in response to the specific needs of these groups, especially when responding to the various and often complex needs of people who experience disadvantage and marginalisation. One method to identify patterns of characteristics within groups is hierarchical cluster analysis [4,5], and this method has been used previously applied to data collected using the Health Literacy Questionnaire which is informed by a strong and comprehensive a priori model of health literacy [6–10].

In general, epidemiological analysis provides information about prevalence and also associations between target variables and independent sociodemographic variables [11–17]. This approach is problematic in health literacy research because health literacy is a multidimensional concept with several independent components where one or more may play a dominant role in outcomes in some contexts but not others [18]. An analytic method such as cluster analysis can complement epidemiological information by analysing health literacy patterns across a range of sociodemographic variables and investigating interactions between these variables [2].

Data from instruments that capture the multidimensional elements of health literacy are now being used for making decisions about public health and policy [19–21]. Importantly, the multiple elements of health literacy have the potential to provide practical guidance for actions to improve public health programs and policies around the world [20]. A globally relevant perspective of health literacy is one that seeks to recognize the diverse ways in which knowledge is produced, transferred, exchanged and used in different countries, cultures and settings around the world, and especially how health knowledge accumulates in families, communities and societies through daily, often communal, activities and social interactions [18]. This global perspective of health literacy recognizes that one-size-fits-all strategies and initiatives to develop health literacy will not be effective in all settings, nor for all people within a setting. Measurement of health literacy in global settings must not only allow for but also look for differences across the multiple dimensions of health literacy [18]. To do otherwise would be to potentially impose dominant culture notions of health literacy where these are not relevant and increase health inequities [3]. The majority of health literacy research has been undertaken in countries such as the USA and Australia [22].

India is a highly diverse country with a rich range of languages, cultures, and geography, and with wide socioeconomic gradients. To date, only a few studies describing health literacy in India have been published (for example, [23–26]) despite wide recognition that it is a useful concept for public health development [19,27]. The study reported in this paper is the first stage of a larger study in a rural area of India. The larger study investigates the use of the community-based participatory Ophelia (Optimising Health Literacy and Access) process [5,6], which is being used to co-design and implement public health initiatives to support medication adherence among people with hypertension.

The focus of this paper is to comprehensively describe the health literacy of the study population using a widely used health literacy questionnaire that was developed in a Western context but has been increasingly tested and applied in non-Western settings. The specific aim of this study is to investigate and compare epidemiological analysis and cluster analysis and explore the utility of each method for describing health literacy strengths and challenges. The study outcomes will inform the next stages of the larger study and are expected to lead to the development of meaningful and context appropriate public health initiatives and policies.

## Materials and methods

### Study design and setting

A cross-sectional survey was conducted in Chandigarh in the north of India, which is a union territory and joint capital for two neighbouring states. The region comprises 22 villages [28]. One village, Faidan Nizampur, in the south of the region, was selected for this study. This rural village was selected purposively because it has a diverse population and is one of the least developed villages in terms of infrastructure. Most of the houses are small and situated in narrow streets with no sealed roads. It has poor sanitation and water supply. Illiteracy in urban areas of Chandigarh is high (14%) and higher in the rural areas (19%) [29]. Faidan Nizampur has 1,072 households and a population of approximately 9,728 people, of which 3,475 are adults.

### Participants

A sample size of 500 was estimated for the main study that will seek to develop community-based interventions to improve medication adherence among people with hypertension. It was expected that this sample size will allow the detection of small differences in health literacy scores between demographic groups, assuming group mean differences between demographic factors of 0.3 and a standard deviation of 0.5 across HLQ scales, with alpha = 0.05 and beta = 0.80. There are no established standards for a minimum sample size for cluster analysis, but previous studies have demonstrated stable cluster analysis with fewer than 500 HLQ respondents [9,30].

There was no existing map of Faidan Nizampur village with marked locations of its households, and this study could not proceed with the systematic random selection of households without such a map. With the help of a geographic information systems (GIS) expert and a field worker, an estimated map of the village with its boundaries, households, and prominent land masses was prepared. First, the field worker surveyed the village and marked village boundaries, including specific places such as temples, clinics, schools, roads, drains, and houses. These data were then provided to the GIS expert who used the information to make the map of the village. Of the 1,072 households identified, 255 households were selected using computer-generated random numbers. It was assumed that two adults would be residing in each household. Adults from each household who were 18 years or over, and who intended to reside in the village for a minimum of one year, were invited to take part in the study. If, after two visits, there was no answer, or the occupants of the selected household chose not to participate, the researchers approached the occupants of the household to the right of the household initially selected.

### Health literacy measurement

The Health Literacy Questionnaire (HLQ) was developed through a grounded validity-driven approach [31], where the constructs were derived directly from the lived experiences of patients and frontline health workers [32]. The HLQ generates information about nine separate health literacy dimensions to provide a profile of the strengths, challenges, and preferences of populations. HLQ data can be used to inform public health planning and evaluation for diverse health literacy needs [5–7]. Validity testing in a wide range of cultural and linguistic contexts provides consistent evidence of good to excellent psychometric properties. The HLQ has been used and tested in Africa [33], Asia [34,35], South America [36], Europe [37–41]; and the Middle East [42].

A key attribute of the measurement theory of the HLQ is the recognition that health literacy is a complex multidimensional concept where different individuals can have different sets of health literacy strengths and challenges (i.e., different health literacy profiles). Health literacy profiles may be similar for people with similar backgrounds, who live in similar contexts, and who have similar lived experiences [43]. Given that different groups of people can have different patterns of health literacy strengths and challenges across the multiple dimensions, a single total score is a poor representation of health literacy diversity. It is for this reason that data from the HLQ are presented as nine separate scale scores to preserve the multiple dimensions and the diverse patterns of health literacy strengths and challenges within a population.

A licence from Swinburne University of Technology in Australia was obtained to use the Hindi version of the HLQ in this study. The HLQ consists of 44 questions within nine conceptually and psychometrically distinct scales:

1. Feeling understood and supported by healthcare providers
2. Having sufficient information to manage my health
3. Actively managing my health
4. Social support for health
5. Appraisal of health information
6. Ability to actively engage with healthcare providers
7. Navigating the healthcare system
8. Ability to find good health information
9. Understand health information well enough to know what to do

HLQ Scales 1 to 5 have response options of ’strongly disagree,’ ’disagree,’ ’agree’ and ’strongly agree’ and a score range of 1 to 4. Response options for Scales 6 to 9 are ’cannot do or always difficult,’ ’very difficult,’ ’quite difficult,’ quite easy’ and ’very easy’ and the score range is from 1 to 5. Scale scores are calculated as per the scoring instructions [32] by averaging the scores of each item within scales. All items have equal weighting.

### Data collection

Community health workers invited eligible people from the randomly selected households to participate. This included providing written and verbal information about the research, obtaining informed consent, and informing them they may stop or withdraw from the study at any time. All adults in a household who consented to participate were administered the questionnaire orally in Hindi. The interview also collected demographic data (see Table 1), including blood pressure (BP, twice at a gap of a minimum of five minutes) using a digital sphygmomanometer. A range of other clinical and anthropometric data were also collected and will be reported in future publications.

**Table 1:** Sociodemographic characteristics.

**Socio- demographic variables**	**Number (%)** 510 (100)
**Sex**	
Women	302 (59.2)
Men	208 (40.8)
**Age**	
18-35 years (young adults)	317 (62.1)
36-55 (middle-aged adults)	160 (31.4)
56-65 (older adults)	25 (4.9)
Above 65 years (elderly)	8 (1.6)
**Religion**	
Hindu	435 (85.3)
Sikh	37 (7.2)
Muslim	33 (6.5)
Christian	5 (1)
**Caste**	
General	202 (39.6)
Other backward class	61 (12)
Scheduled caste	241 (47.2)
Scheduled tribes	6 (1.2)
**Education**	
Illiterate	179 (35.1)
Literate and below class 5	17 (3.3)
Primary school	87 (17.1)
Middle school	96 (18.7)
High or Secondary school	111 (21.8)
Graduate or Post-graduate	19 (3.7)
Professional or Honours	1 (0.2)
**Occupation**	
Unemployed/ home maker	303 (59.4)
Unskilled worker	87 (17)
Semi-skilled worker	84 (16.5)
Skilled worker	8 (1.6)
Clerical, shop owner, farmer	24 (4.7)
Semi- professional	3 (0.6)
Professional	1 (0.2)
**Socio economic class**	
Lower	9 (1.8)
Upper lower	411 (80.5)
Lower middle	85 (16.7)
Upper middle	5 (1)
Upper	0 (0)
**State of origin**	
Uttar Pradesh	235 (46.1)
Bihar	101 (19.8)
Punjab	57 (11.1)
Haryana	48 (9.4)
Himachal Pradesh	9 (1.8)
Chandigarh	43 (8.4)
Other	17 (3.4)

Caste: A social system wherein, the society is hierarchically divided into social categories. It is ascribed status assigned at birth and is closed for mobility.

Socio economic class classification is as per widely accepted scale in India named Kuppuswami scale (modified) 2019 (starting from lower class to upper class).

### Epidemiological analysis

HLQ scale scores for all participants were compared across sociodemographic characteristics, as previously described by Beauchamp et al [43]. Discrete data were presented as proportions while continuous variables were expressed as means, standard deviations (SD) and 95% confidence intervals (CI). With the nine scale scores of the HLQ as dependent variables and sociodemographic characteristics and history of chronic conditions as independent variables, we conducted robust analysis of variance (ANOVA) and a post-hoc test using Games-Howell method where required. Independent variables included male/female; age (18–35 years, 36–55 years, 56–65 years and above 65 years); education (illiterate, literate and below grade 5, primary school, middle school, high or secondary school, graduate or post-graduate, and professional); socio-economic status (SES) using modified Kuppuswamy scale 2019 (lower, upper-lower, lower-middle, upper-middle and upper) [44]; hypertension diagnosed by a physician at least two weeks ago; new case of hypertension (average systolic BP at or above 140 and/or average diastolic BP at or above 90 mm Hg at the time of survey); internal migrant (people coming from other states and residing in the village for livelihood or other reason); having a health card permitting free medical services at a hospital; and self-reported chronic illnesses.

Effect size (ES) using Cohen’s d was used to estimate the strength of the associations between health literacy mean scores and other variables. Cohen’s d is determined by calculating the mean difference between sub-groups and dividing by the pooled standard deviation. ES was assumed to be small if it was from 0.20–0.49, medium if it was from 0.50–0.79, and large if it was >0.80 [45]. Statistical significance was set at p<0.05.

### Cluster analysis

Cluster analysis is an exploratory multivariate method used to identify groups of people with similar patterns of the predefined variables. Cluster analysis does not distinguish between dependent and independent variables and so allows for grouping (or clustering) of all the variables to reveal different patterns within populations. In this study, cluster analysis was used to reveal groups of people who reported similar patterns of health literacy scores across all nine scales, and then to explore related patterns across sociodemographic characteristics and chronic conditions.

The nine mean scale scores from respondents were standardized and converted into Z-scores. Hierarchical cluster analysis was then undertaken using Ward’s method for linkage as recommended by the developers of the Ophelia process [5,7]. Three criteria were used to determine the number of clusters: demographic and clinical variables; distance coefficient in agglomeration schedule; and standard deviation (<0.6 for each scale’s mean score). The sociodemographic variables examined were gender, age, education, occupation, socio-economic status, history of internal migration, clinical variables (chronic illness, previous diagnosis of hypertension, hypertension at time of survey), and the number of people who had free health-cards. Working from 3 through to 16 cluster solutions, the pattern generated by each consecutive cluster split was examined and compared with the parent cluster to identify differences between the patterns until further splits no longer provided new or meaningful patterns in the data.

### Ethical considerations

The study plan was approved by Institute Ethics Committee, Post-Graduate Institute of Medical Education and Research Chandigarh (INT/IEC/2019/000414; Date: 01/03/2019). Informed consent was obtained from all participants and confidentiality was maintained. The study was registered under the Clinical Trial Registry of India (CTRI/2019/10/021827; 31/10/2019).

## Results

### Sociodemographic characteristics of participants

Data from 199 households (510 participants) were included. The household response rate was 76.3% and on 62 (23.7%) of occasions, the adjacent house was approached where a response was collected on 100% of approaches. The overall response rate was 76.2%. Among the 510 participants, 59.2% were women, most (62.1%) were young adults aged 18–35 years and only 1.6% were older than 65 years. Most of the participants were internal migrants (91.6%) and were Hindus (85.3%). Almost half (47.2%) belonged to scheduled caste, a socially disadvantaged class in India. More than one-third of participants were illiterate (35.1%) and only 3.7% were college or university graduates or had obtained further higher education. One-third of participants (33.3%) were unskilled or semi-skilled workers. Most participants belonged to upper-lower class (80.5%) as per Kuppuswami classification of socio-economic status (SES) 2019 [44].

### Epidemiological analysis

**Fig 1:**
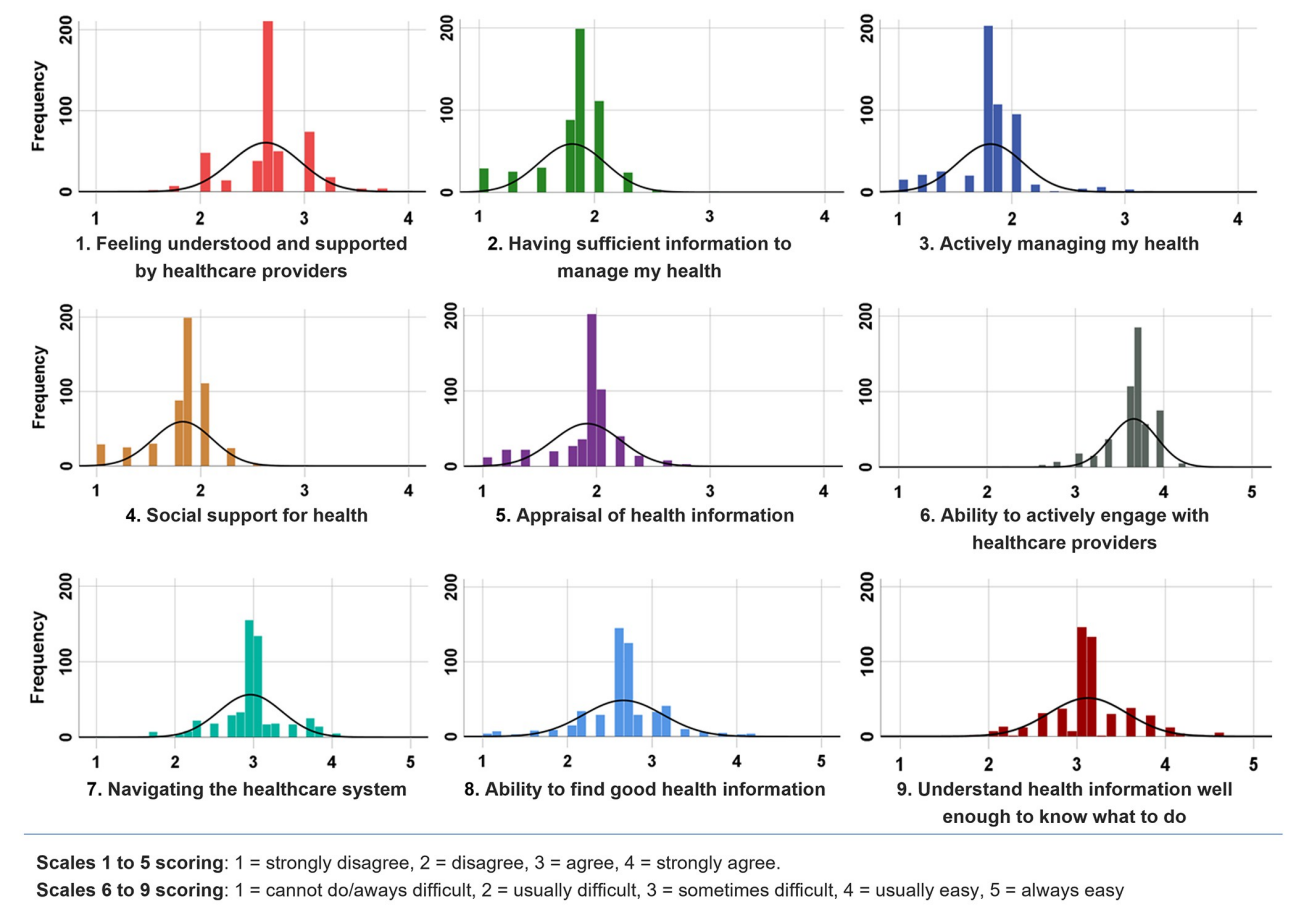
Distribution of scores across the nine health literacy questionnaire scales.

Table 2 displays the mean scores and Fig 1 displays the distribution of mean scores for the nine HLQ scales. Overall, for scales 1 to 5 (score range from 1 to 4), participants reported the highest scores for Scale 4. Social support for health (mean 2.88; SD 0.25), indicating that on average they had good social support. The lowest scores (mean 1.81; SD 0.28) were for Scale 3. Actively managing my health, indicating that many people do not proactively manage their own health care.

**Table 2:** Health Literacy Questionnaire mean scores for the nine scales.

HLQ scale	Mean (SD) [95 % CI]
	Range 1 (lowest) -4 (highest)
1. Feeling understood and supported by healthcare professionals	2.62 (0.33) [2.59, 2.65]
2. Having sufficient information to manage my health	1.80 (0.28) [1.78, 1.83]
3. Actively managing my health	1.81 (0.28) [1.78, 1.83]
4. Social support for health	2.88 (0.25) [2.86, 2.91]
5. Appraisal of health information	1.91 (0.29) [1.89, 1.94]
	Range 1 (lowest) -5 (highest)
6. Ability to actively engage with healthcare professionals	3.66 (0.26) [3.63, 3.68]
7. Navigating the healthcare system	2.95 (0.40) [2.92, 2.99]
8. Ability to find good health information	2.65 (0.46) [2.61, 2.70]
9. Understand health information enough to know what to do	3.12 (0.44) [3.08, 3.16]

There was no missing data.

For Scales 6 to 9 (score range from 1 to 5), the highest reported scores were for Scale 6. Ability to actively engage with healthcare providers (mean 3.66; SD 0.26) and the lowest scores were for Scale 8. Ability to find good health information (mean 2.65; SD 0.46) These scores indicate that, although participants proactively communicated with their healthcare providers, they were experiencing difficulties in finding good health information.

Table 3 provides details about the associations between sociodemographic characteristics and the nine HLQ scales. The largest effect sizes (greater than 1) between demographic groups were observed among age groups for Scale 4. Social support for health, where the youngest group (18–35 years) scored lowest. Also, people with middle SES scored significantly higher than those with lower SES for Scales 7. Navigating the healthcare system, 8. Finding good health information, and 9. Understanding health information well enough to know what to do. Moderate to large differences were observed between men and women in Scales 7. (ES = 0.67), 8. (ES = 0.44) and 9. (ES = 0.53), but lower than women (ES = 0.24) in Scale 6. Ability to actively engage with healthcare provider.

**Table 3:** Association between health literacy and sociodemographic characteristics.

Factors(n)				Part 1			Part 2		
	**1 (HPS)** **Mean (SD)**	**2 (HSI)** **Mean (SD)**	**3 (AMH)** **Mean (SD)**	**4 (SS)** **Mean (SD)**	**5 (CA)** **Mean (SD)**	**6 (AE)** **Mean (SD)**	**7 (NHS)** **Mean (SD)**	**8 (FHI)** **Mean (SD)**	**9 (UHI)** **Mean (SD)**
**Gender**									
Women (302)	2.75 (0.25)	1.81 (0.25)	**1.78 (0.24)**	2.87 (0.28)	1.92 (0.26)	**3.68 (0.19)**	**2.84 (0.28)**	**2.57 (0.38)**	**3.02 (0.35)**
Men (208)	2.63 (0.42)	1.79 (0.33)	**1.84 (0.33)**	2.90 (0.31)	1.90 (0.34)	**3.61 (0.34)**	**3.11 (0.48)**	**2.78 (0.54)**	**3.26 (0.51)**
Difference	0.01	0.02	0.06	0.03	0.02	0.07	0.27	0.21	0.24
ES	0.02	0.04	0.19	0.11	0.03	**0.24**	**0.67**	**0.44**	**0.53**
**Age (in years)**									
18-35 (317)	**2.57 (0.32)**	1.82 (0.28)	1.80 (0.29)	2.66 (0.25)	1.93 (0.29)	3.64 (0.27)	**2.91 (0.30)**	**2.67 (0.46)**	**3.14 (0.44)**
36-55 (160)	**2.71 (0.33)**	1.79 (0.27)	1.83 (0.26)	2.93 (0.25)	1.90 (0.28)	3.07 (0.38)	**3.07 (0.38)**	**2.69 (0.42)**	**3.14 (0.43)**
56-65 (25)	**2.70 (0.40)**	1.65 (0.40)	1.74 (0.37)	2.94 (0.25)	1.90 (0.28)	2.85 (0.42)	**2.85 (0.42)**	**2.34 (0.67)**	**2.91 (0.39)**
>65 (8)	**2.70 (0.15)**	1.82 (0.16)	1.75 (0.18)	2.90 (0.17)	1.74 (0.40)	2.94 (0.31)	**2.94 (0.31)**	**2.37 (0.36)**	**2.62 (0.51)**
Difference (1&2)	0.14	0.03	0.03	0.27	0.03	0.57	0.16	0.02	0.00
ES (1&2)	0.43	0.10	0.10	1.08	0.10	0.11	0.46	0.04	0.00
Difference (1&3)	0.13	0.17	0.06	0.28	0.03	0.21	0.06	0.33	0.23
ES (1&3)	0.35	0.49	0.18	1.12	0.54	0.16	0.16	0.57	0.55
Difference (1&4)	0.13	0.00	0.05	0.24	0.19	0.70	0.70	0.30	0.52
ES (1&4)	0.52	0.00	0.20	1.12	0.07	0.13	0.13	0.72	1.90
**Education**									
1-Illiterate (179)	2.62 (0.32)	**1.73 (0.31)**	**1.71 (0.29)**	**2.83 (0.26)**	**1.84 (0.31)**	**3.62 (0.26)**	**2.77 (0.38)**	**2.40 (0.46)**	**2.87 (0.37)**
2-Below Secondary (104)	2.58 (0.32)	**1.73 (0.31)**	**1.80 (0.26)**	**2.88 (0.26)**	**1.83 (0.30)**	**3.62 (0.27)**	**2.92 (0.35)**	**2.59 (0.36)**	**3.06 (0.29)**
3-Secondary or above (227)	2.65 (0.34)	**1.89 (0.22)**	**1.88 (0.27)**	**2.93 (0.22)**	**2.01 (0.25)**	**3.70 (0.25)**	**3.11 (0.37)**	**2.88 (0.39)**	**3.34 (0.43)**
Difference (1&2)	0.4	0	0.09	0.05	0.01	0	0.15	0.19	0.29
ES (1&2)	0.12	0	0.32	0.19	0.03	0	**0.41**	**0.46**	**0.57**
Difference (1&3)	0.03	0.16	0.17	0.10	1.77	0.08	0.34	0.48	0.47
ES (1&3)	0.09	**0.62**	**0.60**	0.19	**0.60**	**0.31**	**1.21**	**1.12**	**1.17**
Difference (2&3)	0.07	0.16	0.08	0.05	1.78	0.08	0.19	0.29	0.28
ES (2&3)	**0.21**	**0.33**	**0.29**	**0.20**	**0.65**	**0.30**	**0.90**	**0.79**	**0.76**
**Socio-economic class**									
Lower (420)	2.61 (0.31)	**1.78 (0.28)**	**1.78 (0.28)**	**2.86 (0.24)**	**1.88 (0.29)**	3.64 (0.26)	**2.87 (0.35)**	**2.57 (0.41)**	**3.02 (0.38)**
Middle (90)	2.68 (0.41)	**1.92 (0.25)**	**1.93 (0.27)**	**2.97 (0.26)**	**2.07 (0.27)**	3.70 (0.27)	**3.34 (0.38)**	**3.07 (0.46)**	**3.57 (0.42)**
Difference	0.07	0.14	0.15	0.11	0.19	0.06	0.47	0.50	0.55
ES	0.19	**0.52**	**0.52**	**0.43**	**0.67**	**0.22**	**1.28**	**1.14**	**1.37**
**Internal migration**									
Yes (467)	2.63 (0.34)	**1.79 (0.29)**	1.80 (0.29)	2.85 (0.26)	**1.90 (0.29)**	3.65 (0.29)	2.95 (0.40)	2.64 (0.46)	3.11 (0.43)
No (43)	2.57 (0.28)	**1.91 (0.29)**	1.84 (0.26)	2.92 (0.15)	**2.01 (0.27)**	3.69 (0.27)	2.98 (0.33)	2.78 (0.45)	3.18 (0.53)
Difference	0.06	0.12	0.04	0.07	0.11	0.04	0.03	0.16	0.07
ES	0.19	**0.45**	0.14	0.18	**0.39**	0.14	0.08	**0.30**	0.14
**Chronic Illness**									
Yes	**2.71 (0.28)**	1.84 (0.25)	1.85 (0.35)	2.88 (0.24)	1.94 (0.26)	**3.72 (0.24)**	2.95 (0.39)	2.63 (0.46)	3.09 (0.44)
No	**2.60 (0.34)**	1.79 (0.29)	1.79 (0.26)	2.88 (0.27)	1.90 (0.30)	**3.64 (0.26)**	2.95 (0.40)	2.66 (0.46)	3.13 (0.44)
Difference	0.11	0.05	0.06	0	0.04	0.08	0	0.03	0.04
ES	**0.35**	0.01	0.18	0.01	0.11	**0.33**	0.002	0.06	0.08
**Health-card**									
Yes (45)	**2.89 (0.31)**	1.79 (0.33)	**1.90 (0.30)**	**3.01 (0.25)**	1.88 (0.32)	3.67 (0.28)	**3.25 (0.40)**	2.79 (0.52)	**3.27 (0.44)**
No (465)	**2.60 (0.32)**	1.80 (0.28)	**1.80 ((0.28)**	**2.87 (0.25)**	1.91 (0.29)	3.65 (0.26)	**2.93 (0.38)**	2.64 (0.46)	**3.10 (0.43)**
Difference	0.29	0.01	0.10	0.14	0.03	0.02	0.32	0.15	0.17
ES	**0.92**	0.03	**0.34**	**0.56**	0.09	0.07	**0.82**	**0.30**	**0.39**

Means in bold are significant having p<0.05 for difference in mean (tested using robust ANOVA); ES (Effect size) calculated using Cohen’s d for standardized difference in means. ES value >0.20–0.50 is ‘small’, 0.50–0.80 is ‘medium’ and >0.80 is ‘large’; all denoted by bold numbers.

Education was clearly associated with health literacy in all scales, where people with secondary education scored higher than those who did not complete secondary education. ES ranged from 0.20 for Scale 4. to 0.90 for Scale 7. SES was most strongly associated with ability to navigate the healthcare system (Scale 7), as well as finding and understanding health information (Scales 8 and 9), but not for Scale 1. Feeling understood and supported by healthcare providers. Participants who had immigrated from other states scored lower than non-migrants in Scale 2. Having sufficient information to manage health (ES = 0.45), and Scales 5. Appraisal of health information (ES = 0.39) and 8. Ability to find good information (ES = 0.30).

People with a history of a chronic condition reported better healthcare provider support (Scale 1) and better ability to actively engage with their healthcare provider (Scale 6) than those who were not sick (ES 0.35 and 0.33, respectively). People with a health-card had substantially higher health literacy across several scales. They scored much higher (ES = 0.93) in Scale 1. Feeling understood and supported by healthcare providers and had better healthcare navigation skills (Scale 7) (ES = 0.81) than those who did not have a health-card. Also, they had better social support (Scale 4) (ES = 0.56) and skills to find and understand health information to manage health (ES = 0.30 and ES = 0.39 in Scales 8 and 9, respectively).

### Cluster analysis

Cluster analysis identified eight health literacy profiles in the study population (Table 4). Clusters were given designations from A to H. The number of people in each cluster varied from 9 (cluster G) to 276 (cluster C), with characteristics described below.

**Table 4:** Cluster analysis showing eight health literacy clusters and associated demographic characteristics.

Cluster	A	B	C	D	E	F	G	H
Number of participants n (%)	14 (2.7)	54 (10.6)	276 (54.1)	40 (7.8)	50 (9.8)	49 (9.6)	9 (1.8)	18 (3.5)
**Within cluster HLQ mean score (SD)**								
1. Feeling understood and supported by healthcare providers	2.07 (0.15)	3.00 (0.25)	2.64 (0.09)	3.03 (0.17)	2.21 (0.36)	2.54 (0.23)	3.11 (0.45)	2.07 (0.52)
2. Having sufficient information to manage my health	2.09 (0.29)	2.01 (0.13)	1.87 (0.07)	1.33 (0.24)	1.78 (0.28)	1.95 (0.33)	1.17 (0.28)	1.14 (0.20)
3. Actively managing my health	2.03 (0.25)	2.13 (0.31)	1.83 (0.07)	1.63 (0.30)	1.96 (0.37)	1.65 (0.26)	1.18 (0.27)	1.17 (0.18)
4. Social support for health	3.14 (0.17)	3.09 (0.19)	2.89 (0.05)	3.10 (0.23)	2.66 (0.37)	2.85 (0.15)	3.16 (0.17)	2.20 (0.39)
5. Appraisal of health information	2.31 (0.37)	2.10 (0.21)	1.97 (0.06)	1.46 (0.30)	1.84 (0.27)	2.12 (0.22)	1.24 (0.17)	1.16 (0.15)
6. Ability to actively engage with healthcare providers	3.90 (0.15)	3.87 (0.18)	3.66 (0.11)	3.69 (0.25)	3.37 (0.37)	3.92 (0.16)	3.27 (0.37)	3.13 (0.28)
7. Navigating the healthcare system	3.60 (0.29)	3.55 (0.28)	2.98 (0.09)	2.95 (0.38)	2.86 (0.37)	2.57 (0.28)	2.26 (0.30)	2.02 (0.49)
8. Ability to find good health information	3.76 (0.32)	3.17 (0.30)	2.68 (0.11)	2.41 (0.39)	2.76 (0.48)	2.38 (0.36)	1.33 (0.22)	1.59 (0.46)
9. Understand health information well enough to know what to do	4.03 (0.37)	3.67 (0.33)	3.11 (0.14)	3.15 (0.48)	3.22 (0.53)	2.62 (0.36)	2.31 (0.15)	2.48 (0.44)
**Sociodemographic characteristics**								
Mean age (years)	27.7	40.7	33.6	38.2	32.1	35.3	44.6	30.3
Women (%)	7.1	14.8	76.1	40.0	26.0	83.7	55.6	44.4
Illiteracy (%)	0.0	1.9	40.9	27.5	18.0	46.9	100.0	72.2
Secondary education or higher (%)	85.7	85.2	41.3	35.0	48.0	32.7	0.0	5.6
Occupation (%)								
Unemployed	14.2	16.6	67.7	55	38	89.7	77.8	72.3
Clerical/ shop owner/ farmer	0	7.4	1.4	0	0	0	0	0
Average Income	4.3	3.6	3.2	3.5	3.3	3.1	3.9	3.2
Average Socio-economic status	2.9	2.6	2.1	2.2	2.2	2.0	2.0	1.9
Migration (%)	85.7	96.3	88.0	97.5	94.0	98.0	100.0	94.4
Health card (%)	7.1	27.8	5.1	22.5	6.0	2.0	22.2	0.0
**Medical conditions**								
Any chronic illness (%)	7.1	31.5	23.6	15.0	18.0	30.6	22.2	16.7
Known hypertension (%)	7.1	18.5	15.6	10.0	8.0	26.5	11.1	11.1
New hypertension case (%)	42.9	31.5	17.8	20	30	10	11	22

Notes

Colour coding of cells within clusters are based on green (highest) and red (lowest) health literacy. Scores are only relative to other levels in the row and do not indicate predetermined high/low scores related to each scale.

**Average income**: 1- monthly income equal or less than Rs.2,640, 2- Rs.2,641–7,886, 3- Rs.7,887–13,160, 4- Rs.13,161–19,758, 6- Rs.19,759–26,354, 10- Rs.26,346–52,733 and 12- more than Rs.52,734.

**Average socioeconomic status**: 1 lower, 2 upper-lower, 3 lower-middle, 4 upper-middle class.

**Known hypertension**: Person previously diagnosed with hypertension by a physician for at least past 2 weeks prior.

**New hypertension case**: Detected hypertension during the interview.

This was the second smallest cluster of younger adults, mainly men, who were educated and had a good income. They scored highest among all clusters in Scales 8. Ability to find good information (mean 3.76) and 9. Understand health information well enough to know what to do (4.03), and lowest in Scale 1. Feeling understood and supported by a healthcare professional (2.07). This cluster had the highest rate of new cases of high blood pressure (42.9%) and most were unaware they had this health condition.

***Cluster B*:** *Have knowledge of healthcare resources and support from healthcare providers but lack good health information* (n = 54)

This cluster constituted mainly middle-aged males (mean 40.7 years). Almost one-third (31.5%) had a chronic illness and equal proportion were found to have previously unknown high blood pressure. More of these participants had health-cards than in any other cluster. They were educated but did not think they had enough information, as indicated by the mean score for Scale 2. Having sufficient information to manage own health (2.01).

***Cluster C*:** *Have the ability to understand health information well but have support from a healthcare provider and do not actively manage own health* (n = 276)

This was the largest cluster, mainly comprised of young adult women (mean age 33.6 years). Many of them were illiterate (40%) with about two-third (67.7%) unemployed. Scores in Scale 1 (2.61) indicated the people in this cluster don’t always feel understood and supported by healthcare providers and the low mean score (1.87) in Scale 3 suggests they were not spending much time actively managing their health.

**Cluster D:** *Have good support from healthcare providers but limited ability to find or appraise health information* (n = 40)

The people in this cluster (men 60%; women 40%) had low education (only 35% had completed secondary education or higher) and had middle range incomes. They felt they didn’t have adequate information, as shown by a very low score (1.33) for Scale 2. Having sufficient information to manage health, despite having good support from their healthcare providers (Scale 1 mean score of 3.03).

**Cluster E:** *Low support from healthcare providers and limited ability to engage with them* (n = 50)

People in this cluster were mostly young adult men (74%), with almost half having completed secondary education and who could generally understand health information (Scale 9 score of 3.22). However, they felt they did not have adequate health information to manage their health (score of 1.78 for Scale 2. Having sufficient information to manage health). They also felt they had little support from a healthcare provider (Scale 1 score of 2.21) and were not confident in actively engaging with them (Scale 6 score of 3.37).

**Cluster F:** *Highest ability to engage with healthcare providers but do not have sufficient information and are not managing their own health* (n = 49)

The people in this cluster were mostly women (83.7%), of whom almost half were illiterate and with low SES. This cluster had the highest number of participants with chronic conditions (30.6%) and a low score (1.65) for Scale 3. Actively managing my health. They could, however, communicate well with doctors and scored the highest among all clusters (3.92) for Scale 6. Ability to actively engage with healthcare providers.

**Cluster G:** *Good social and healthcare provider support but limited access to and understanding of health information* (n = 9)

This was a small group of people, but this cluster had the highest average age (44 years) among all clusters. All participants were illiterate migrants belonging to low SES, and 22% had health-cards. These participants reported having good healthcare support (Scale 1 score of 3.11) and social support (Scale 4 score of 3.16). However, they were experiencing challenges in all other HLQ dimensions.

**Cluster H:** *Limited healthcare and social support with inadequate understanding of health information* (n = 18)

This was a cluster with younger people (mean age 30.3 years) and almost three quarters (72.2%) were illiterate. No participant reported having a health-card. Unlike other clusters, which generally had a higher score for social support (Scale 4), this cluster reported the lowest social support among all clusters (2.20). In fact, this cluster also had the lowest scores among all clusters for all scales except Scale 1 (2.07), which had the same scores as Cluster A (2.07).

## Discussion

In this study we conducted standard descriptive analysis and cluster analysis to explore the health literacy, sociodemographic, and other health-related characteristics of people living in a resource poor context in northern India. We sought to generate a nuanced understanding of what the data mean to inform the development of an intervention strategy to address the health literacy needs of this rural population. The study also allowed for comparison of methods by which data can be interpreted and how those interpretations may influence decisions about public health initiatives and policies.

The epidemiological analysis examined overall population health literacy mean scores and indicated that, on average, most participants in this study had multiple health literacy challenges, except in the areas of having social support for health and their ability to engage with and feel supported by their healthcare providers. Using this method of data analysis, decisions might be made about population-level initiatives to support people to navigate the healthcare system, to actively manage their health, to understand and appraise health information, and to help people to find and have sufficient health information.

Cluster analysis examined the health literacy patterns among participants and related these patterns to demographic and health-related data, which resulted in eight groups (clusters) of people with distinct patterns of demographic, health condition, and health literacy characteristics. Importantly, this analysis uncovered a group of young men with relatively few health literacy challenges but with high rates of undiagnosed high blood pressure. While the descriptive analysis showed that this population, overall, has health literacy challenges, the cluster analysis revealed sub-groups of people with similar demographics and health conditions who have specific patterns of health literacy strengths, challenges and preferences. Cluster analysis, although not commonly used in public health, has the potential to inform the development of public health initiatives that are tailored to specific population subgroups, especially groups that frequently experience disadvantage or marginalisation [6,8,46–48].

### Descriptive epidemiological analysis and health literacy needs

In public health research, descriptive epidemiological analysis is useful for studying disease distribution, exploring risk-factors of a disease, and identifying public health hazards and other factors [49]. Score distributions were examined in this study in India and indicated that there was a wide distribution of health literacy across the population (Fig 1), and most people appeared to have several health literacy challenges. The epidemiological analysis provided the average health literacy scores and associations with other characteristics of the population, and this helped to draw attention to some key characteristics, such as people generally having good social support for health, which suggests these are potential strengths that could be built on. Importantly, although most people in this study were migrants, and some were living alone, the majority reported having good social support for health in their community. On average, the data indicated that people also had good support from at least one healthcare provider and were able to actively engage with them (Scales 1 and 6).

The epidemiological analysis provided information on some health literacy challenges experienced by the participants. For example, most participants who did not have secondary education reported markedly lower scores in Scales 5, 7, 8, and 9, which concurs with the results of other studies where education levels were found to be associated with health literacy [6,50]. Similarly, low SES was associated with lower scores for all health literacy scales except for Scale 1. Feeling understood and supported by healthcare providers [51]. Being male seems to have a role in enabling access to health services because men tended to have higher scores than women in scales relating to navigating the healthcare system and finding and understanding health information. This is expected because many women have reduced access to resources in this population [52,53].

Having a health-card permitting free treatment was associated with people who had a relatively good ability to navigate the healthcare system, felt understood and supported by healthcare providers, and had the ability to get good health information and understand information to manage their health. Studies have shown that patients who had opportunities to avail low-cost or free treatments had better chronic disease management, therefore, such facilities are necessary for supporting health as well as promoting health literacy, especially for low SES populations [54].

### Clusters and health literacy needs

The descriptive epidemiological analysis investigated average associations between the overall population health literacy scores and individual characteristics. Cluster analysis extended this investigation to uncover eight groups of people with different patterns of specific health literacy strengths and needs and related sociodemographic and clinical characteristics. Specific needs, especially for groups that experience disadvantage or marginalisation, are often masked when investigations only go as far as using average population scores to describe a population’s characteristics. For example, Cluster A had people who were generally well educated, male, with a higher SES, and with HLQ scores that were higher on most scales than other clusters, but scores were surprisingly low in Scale 1. Feeling understood and supported by a healthcare provider. It could be that they were young, considered themselves healthy, and did not see the need to have a regular healthcare provider. Therefore, a striking finding was that almost half of the men in this group were not aware, at the time of data collection, that they had high blood pressure and were in urgent need of active care. This finding demonstrates that efforts are required to uncover the differences among groups within populations so that initiatives can be tailored to meet the different needs of people in different contexts.

Cluster B, which consisted mainly of educated men with a chronic illness and a health-card, had good navigation skills and support and understanding from their healthcare providers. Conversely, although Cluster C had a somewhat similar cluster pattern (but with lower scores), it was by far the largest group and primarily comprised lower educated women. They reported low support from healthcare providers and struggled to navigate the healthcare system. The different education levels of people in the clusters might play a role in this inequity, but gender-based differences could also be a reason for women having lower health literacy. Inequitable access to health care for women is evident in India [55]. This finding calls for interventions to change systems and cultural norms to support women and promote equal access.

People in Cluster D had good support from healthcare providers and some education, but they reported low scores in dimensions linked to health information and they were not able to manage their health well. This finding suggests that people in this cluster, while having some good skills and support, struggle to find health information and understand how to use it to manage their health. The role of service providers is crucial in such circumstances to provide the necessary information in accessible and understandable formats [56].

People in Cluster E (primarily younger men with some education) tended not to engage and had limited ability to engage with healthcare providers and did not report having health conditions. However, 30% of the men in this cluster were found to have high blood pressure at the time of the study. Health check-ups, including blood pressure monitoring, are necessary to detect disease early and would be an essential health promotion program for this group [57,58].

Cluster G was a small, somewhat older, and illiterate group of people who reported very low health literacy on most scales except social support for health and feeling understood by healthcare providers. Only a quarter of this group had a health-card. However, the positive engagements with healthcare providers may be a key strength that could be leveraged to support this group of people to better understand and manage their health.

### Using epidemiological or cluster analysis to inform intervention studies

The epidemiological analysis provided confirmation of associations that are already known: i.e., low education and low SES are associated with health literacy. Effect sizes revealed new information about the relative magnitude of these associations and indicated priority areas in which population health literacy could be improved. In this study context, access to health services could be improved by better understanding and generating system and policy initiatives to change how being a woman, having limited education, and having low SES result in health inequities. However, it is known that these sociodemographic factors are embedded structural inequities in many societies, that they are related to health literacy challenges, and that one-size-fits-all solutions are often employed but rarely lead to systemic and long-lasting changes in health equity. What is needed is to get beyond the known generalisations and to reveal the nuances of needs in different groups within populations.

The cluster analysis results reveal a diverse range of health literacy strengths and needs of groups of people in populations. This means that fit-for-purpose interventions can be developed for each group rather than a generalised whole population intervention. For example, programs, including using digital initiatives, could be developed for and with young people to motivate and support them to go for regular health check-ups, even if they feel well (Clusters A, C, E and H). Also, local healthcare services and community health workers could join with women’s groups to develop ways in which to make healthcare more accessible for women in Cluster C, and other women like them. Provision of door-to-door health information in a range of communication formats to inform and motivate people, such as those in Clusters F and H, would assist them in their self-care. People in Clusters A and B may also benefit from provision of tailored health information and self-care motivation skills training to enable them to use healthcare services to help manage and control their hypertension. Some groups of people may respond well to healthcare providers using communication methods, such as teachback [59,60], when delivering health information to patients, so health literacy responsiveness training for healthcare providers may support better interactions with patients and community members. These interventions mostly include actions that are required from service providers, so they are better able to reach out and respond to the needs of the people they serve, especially the people who experience disadvantage and marginalisation. Government funded health programs should be designed to not leave anyone behind and should therefore be able to reach the unreached, irrespective of societal, physical, and environmental barriers.

This study found that cluster analysis is useful for going beyond generalisations about populations to identify specific strengths and needs of the diverse groups of people within a population. People with different patterns of strengths and needs may need support to overcome barriers to accessing health services in ways that are different to people with other strengths and needs. Cluster analysis extends descriptive epidemiological analysis to reveal the different needs of different groups so that interventions can be tailored to promote health and equity to people in all their diversity.

### Strengths and limitations

A strength of the study is that it was population-based and reached many people who are usually not included in research studies. The study was conducted in a rural setting with a large and young migrant population. Compared with the general population, there was a slight underrepresentation of people older than 60 years (5.6%), which is somewhat lower than the general population (>8%) [61]. We administered the HLQ using one-on-one interviews to promote a high response rate among people who were illiterate. While this increased the generalizability of the findings to this particular village setting, a possible limitation is that interviewer administration may result in bias related to people providing socially desirable responses.

## Conclusion

Descriptive epidemiological and cluster analysis methods were used to explore health needs among residents of a rural north Indian village. The utilisation of population averages, without careful consideration of disparate demographic groups, might lead to the neglect of population subgroups who need tailored solutions to address their needs. This study demonstrates how cluster analysis is useful to expand the detail of epidemiological analyses to inform intervention development by generating nuanced evidence-based profiles across a whole community such that public health planners have actionable data on health literacy strengths and needs so that no group is left behind.

## Supporting information

S1 Data (XLSX)
